# Commentary: The Problem of Mental Action: Predictive Control Without Sensory Sheets

**DOI:** 10.3389/fpsyg.2018.01291

**Published:** 2018-07-25

**Authors:** Giovanni Pezzulo

**Affiliations:** Institute of Cognitive Sciences and Technologies, National Research Council, Rome, Italy

**Keywords:** mental action, predictive processing, active inference, control theory, action-oriented cognition

Action-oriented and pragmatic views of cognition, which propose that the action system is part and parcel of various cognitive functions (e.g., perception, memory, and decision-making), are increasingly popular in philosophy, psychology, neuroscience, and robotics (Engel et al., 2016). Different theories stress distinct aspects of action-directedness, such as for example the importance of sensory-motor regularities or contingencies to steer active perception loops (O'Regan and Noe, 2001; Ahissar and Assa, 2016); the reuse of the brain's motor system for “action simulation,” in the service of action perception, imagery, and planning (Jeannerod, 2006); that the brain may be organized to rapidly specify and select actions (Cisek, 1999; Cisek and Kalaska, 2010; Pezzulo and Cisek, 2016). There is however one aspect of action-directedness that has received less attention so far: the possibility for cognitive agents to perform *mental actions*.

Unlike physical actions, mental actions do not modify the external environment directly. Yet, some internal cognitive operations can be conceptualized as (mental) *actions* in virtue of their intentional or purposive structure; or, in other words, because they (are selected to) achieve some form of outcome or goal—even if the goal is not specified in terms of outward behavior. Metzinger proposed to conceptualize mental actions as internal operations that change a cognitive agent's state of knowledge (Metzinger, 2017). Mental actions thus have *epistemic goal-states* (e.g., knowing what the sum of 2 + 3 is) as opposed to the most usual *pragmatic goal-states* (e.g., reaching a goal location). According to (Metzinger, 2017, p. 1): “*Examples of mental action are the volitional control of endogenous attention […], trying to retrieve a series of images from episodic memory, […] as well as engaging in mental calculation, […]*.”

The idea that mental actions serve epistemic purposes prompts many questions about their role in the architecture of cognition. Here, I focus on a specific question: when should a cognitive agent select a mental action as opposed to a physical action? I will address this problem by casting the above definition of mental action within Active Inference (Friston, 2010; Friston et al., 2012, 2015; Pezzulo et al., 2015, 2017a, 2018).

A tenet of Active Inference is that the brain encodes statistical regularities at multiple timescales—in the form of *internal generative models*—to steer top-down predictions that support both perception and action. For example, during action selection, the internal models are used to predict and compare the outcomes of alternative courses of actions (or policies). Importantly, in Active Inference, policy evaluation resolves the exploration-exploitation tradeoff by considering both the *pragmatic* and the *epistemic* value of actions. With some simplifications, one can imagine two kinds of actions or policies: those that have mainly pragmatic value (exploitation; e.g., navigating to a reward site) and those that have mainly epistemic value (exploration; e.g., collecting a cue to resolve one's own uncertainty about reward location). Computational simulations of foraging show that, during uncertain choices, an agent often needs to firstly select an *epistemic action* to resolve its uncertainty (e.g., go to a cue location, to collect information about reward location), before it can commit to a specific choice, i.e., before it can confidently select a *pragmatic action* that leads to the disambiguated reward location (Friston et al., 2015; Pezzulo et al., 2016).

Interestingly, mental actions may be treated in the same way as overt, exploratory actions (e.g., collecting cues) in these foraging simulations; by considering that both serve the epistemic goal to reduce one's own uncertainty about a variable of interest (e.g., uncertainty about “what the sum of 2 + 3 is” vs. “where the reward location is”). Indeed, the exploration-exploitation tradeoff illustrated in the foraging simulations arises in many other choice situations that may involve both physical and mental actions. Imagine one is checking out an apartment for rent, and has to leave the keys inside. This situation may involve the competition between various policies: a policy that only includes *pragmatic actions*, and which consists in closing the door immediately; a policy that includes *mental epistemic actions*, and which consists in carefully double-checking mentally whether one has collected all the necessary luggage, until one is confident enough and can then close the door; and a policy that includes *physical epistemic actions*, and which consists in visiting once more all the rooms, before closing the door. Clearly, policy selection would involve various kinds of trade-offs, both between pragmatic vs. epistemic actions (e.g., a “mental check” takes time but lowers the risk of forgetting luggage), and between mental vs. physical epistemic actions (e.g., a “mental check” is usually faster but more error-prone compared to physical search).

However, these are exactly the sort of trade-offs that arise in any instance of Active Inference, which automatically balances pragmatic and epistemic aspects of policies, depending on various factors such as uncertainty, time pressure and the importance of current goals. In other words, the selection of a mental action (e.g., tapping an episodic memory or performing mental arithmetic) that changes one's epistemic state (e.g., reduces uncertainty before a choice) would conform to the usual exploration-exploitation tradeoffs that have been studied in simpler foraging situations (Friston et al., 2015; Pezzulo et al., 2016). Developing a comprehensive theory of mental action within Active Inference would require endowing an agent with internal models of (and epistemic goals about) it's own inference and belief state; for example, models that describe how the agent's belief state would change as an effect of mental actions, and which would permit considering their costs and benefits during policy selection.

This conceptualization casts mental action within a deliberative scheme, as a form of *controlled* activity, where the controlled variables are epistemic goal states and mental processes—e.g., the control of imagination (Pezzulo and Castelfranchi, 2009) or of spontaneous mental behavior (Pezzulo et al., 2014, 2017b; Metzinger, 2017)—as opposed to external variables. In this perspective, even though mental actions don't directly influence the sensory-motor stream, they may still have a causal role in the architecture of cognition (Metzinger, 2017); for example, by altering levels of certainty or confidence in adaptive ways before a difficult choice.

## Author contributions

The author confirms being the sole contributor of this work and approved it for publication.

### Conflict of interest statement

The author declares that the research was conducted in the absence of any commercial or financial relationships that could be construed as a potential conflict of interest.
